# Using spatial and temporal modeling to visualize the effects of U.S. state issued stay at home orders on COVID-19

**DOI:** 10.1038/s41598-021-93433-z

**Published:** 2021-07-06

**Authors:** Rachel Carroll, Christopher R. Prentice

**Affiliations:** 1 Department of Mathematics and Statistics, University of North Carolina Wilmington, Wilmington, NC 28403 USA; 2 Department of Public and International Affairs, University of North Carolina Wilmington, Wilmington, NC 28403 USA

**Keywords:** Software, Statistics, Computational biology and bioinformatics

## Abstract

Coronavirus disease 2019 dominated and augmented many aspects of life beginning in early 2020. Related research and data generation developed alongside its spread. We developed a Bayesian spatio-temporal Poisson disease mapping model for estimating real-time characteristics of the coronavirus disease in the United States. We also created several dashboards for visualization of the statistical model for fellow researchers and simpler spatial and temporal representations of the disease for consumption by analysts and data scientists in the policymaking community in our region. Findings suggest that the risk of confirmed cases is higher for health regions under partial stay at home orders and lower in health regions under full stay at home orders, when compared to before stay at home orders were declared. These results confirm the benefit of state-issued stay at home orders as well as suggest compliance to the directives towards the older population for adhering to social distancing guidelines.

## Introduction

The coronavirus disease 2019 (COVID-19) began in Wuhan, China in December of 2019 and quickly evolved into a global crisis. As of May 18, 2020, data suggested that the pandemic resulted in 1,492,822 confirmed cases and 89,101 deaths in the United States alone^1^. Due to the widespread nature of this crisis and the need to develop data-driven insights into COVID-19 and its spread, data from various reputable sources were made publicly available and updated daily.

In this project, data science tools are coupled with robust statistical modeling to offer interactive real-time exploration and visualization of COVID-19 both regionally and nationally^2^. Data visualization dashboards furnished an ideal environment for understanding the distribution and characteristics of COVID-19 across the United States over time. These dashboards allowed for the easy dissemination of information to a range of audiences in academe, government and nonprofit organizations, and members of the general populace. Supporting code and links to these dashboards as well as an array of related visualizations created by colleagues in the UNCW Data Science program can be found here.

The first steps of this project involved compiling data from multiple sources, including: COVID-19 data from Johns Hopkins University Center for Systems Science and Engineering^1^, population and demographic information from the US Census Bureau, airport and travel data from the US Transportation Department and US Bureau of Transportation Statistics, and unemployment information from the US Bureau of Labor Statistics^3–6^. The stay at home orders variable was self-constructed with guidance from government mandates and the New York Times^7^. After compiling the data, we constructed visualization dashboards for optimal viewing, focusing on those values that vary over time (e.g., confirmed COVID-19 cases). Next, we implemented the statistical model to spatially and temporally explore the prevalence of confirmed cases and death outcomes. The statistical model we employed was a Bayesian spatio-temporal Poisson disease mapping model that allowed for estimation of important fixed effect parameter estimates in addition to residual spatial and temporal variation^8–10^. The dashboards linked above display the real-time implementation of this statistical model. The results reported in this article focus on the time of the pandemic when stay at home orders were largely in effect.

## Results

An R Shiny COVID-19 regression dashboard with the results for the data presented in this manuscript is available here. Data and analysis are constrained to focus on the March 23, 2020 to May 18, 2020 window.

**Figure 1:**
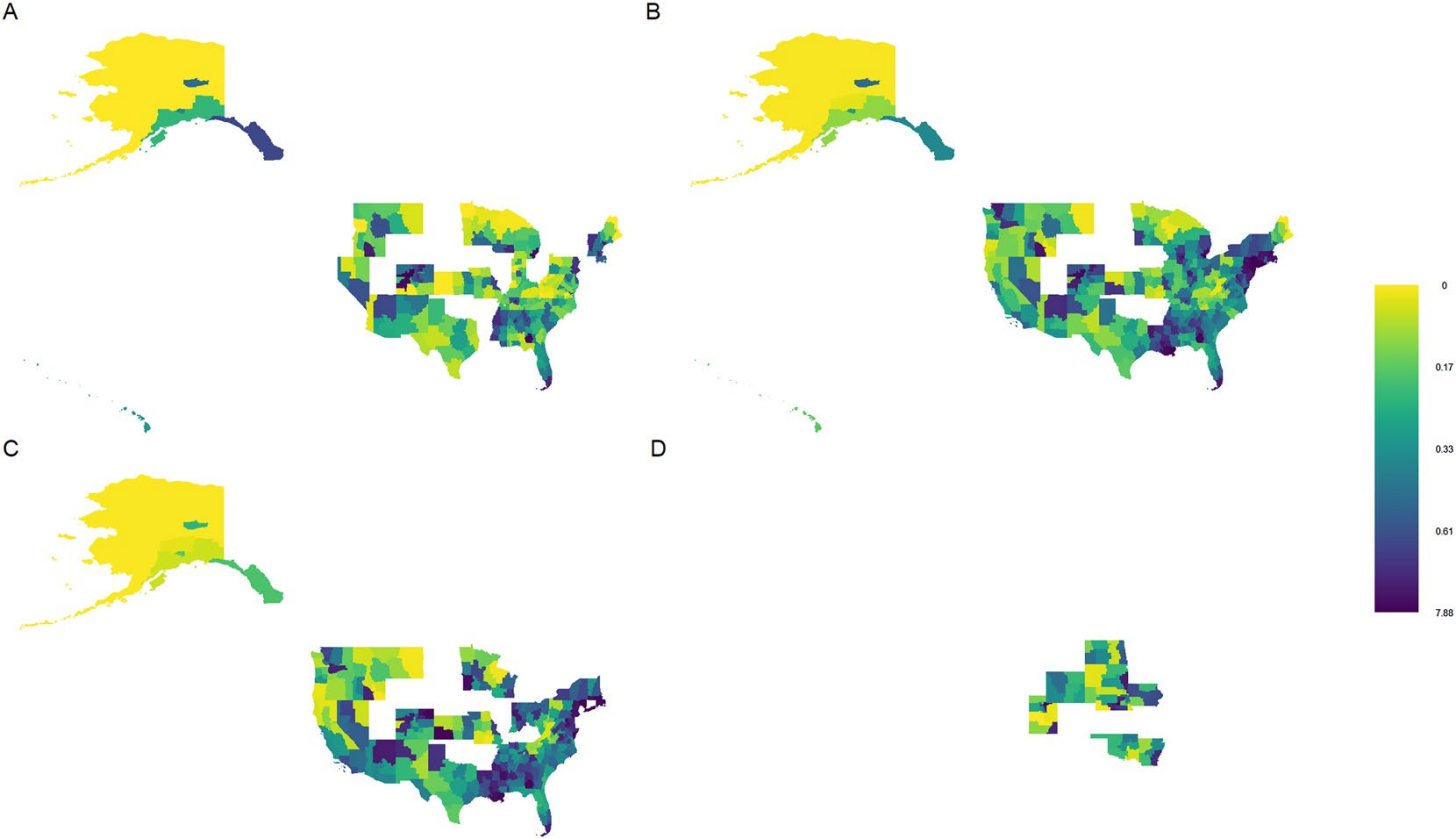
A combination of the stay at home orders variable and standardized incidence ratios by health region for the confirmed cases outcome. (**A**) Before stay at home orders, (**B**) During full stay at home orders, (**C**) During partial stay at home orders, and (**D**) Never stay at home orders. Health regions are only present in panes where the stay at home orders category applied.

Figure 1 displays a combined visualization of the stay at home orders and a calculation of the median standardized incidence ratio (${y}_{ij}/{e}_{ij}$) of confirmed cases for each health region across the days that they were in the given stay at home orders category (before, during—full, during—partial, and never). The same image is available for the deaths outcome in the Supplemental Figures as SAH SMR.png. Figure 1 illustrates the areas of the country where the different levels of the stay at home order exist, and suggests higher risk for areas under partial stay at home orders (darker shading indicates more risk). Medians of these medians represent risk across each category of stay at home orders, and are 0.23 (before stay at home orders), 0.33 (during full stay at home orders), 0.41 (during partial stay at home orders), and 0.25 (never stay at home orders). Results suggest most risk under partial stay at home orders.

An animation of the state-level stay at home orders variable (our main covariate of interest) and the individual images used to create the animation are provided in the supplemental materials as sah.gif and “SAH” (folder), respectively. This animation shows most of the country shut down and then gradually begin to reopen over the time considered in this study. Several states never issued statewide stay at home orders variables—Arkansas, Iowa, Nebraska, North Dakota, Oklahoma, South Dakota, Utah, and Wyoming. The Supplemental Figures in the “SIR SMR” folder display the standardized incidence or mortality ratios for confirmed cases, active cases, new cases, and deaths for each day across all health regions in the United States. Standardized incidence and mortality ratios suggest that the spatial distribution of increased (greater than 1) vs. decreased (less than 1) risk remains largely consistent across time, which provides justification for the separate spatial and temporal random effect terms in the model described above. Despite this relative consistency, there is a slight increase in risk, particularly with respect to death, over time.

Table 1 displays the statistical model fixed effect parameter estimates associated with the confirmed case and death outcomes for the US as a whole and for the four separate US Census-defined regions. An inverse logarithm was applied to these parameter estimates to offer an interpretation directly related to the multiplicative change in relative risk (${\theta }_{ij}$) of the outcome considered. Consequently, an estimate with a 95% credible interval less than one suggests decreased risk while an estimate with an interval greater than one suggests increased risk. The estimates for the other two outcomes—active cases and new cases—are largely consistent across all models (these findings are made available in the linked Shiny dashboard and Supplemental Table 1). Generally speaking, the parameter estimates for the outcomes in Table 1 suggest increased risk under partial stay at home orders and decreased risk under full stay at home orders compared to before the stay at home orders were instated (darkest gray highlighted estimates). Specifically, the estimate from the South confirmed cases outcome model suggest a 5.2% higher risk in health regions under partial stay at home orders and a 5.0% lower risk in health regions under full stay at home orders. Risk appears to decrease as the percent unemployment in a region rises (medium gray highlighted estimates); the estimate suggests that for each one percent increase in unemployment, the risk of confirmed cases increases by 2.2% in the South confirmed cases outcome model. Similarly, one finding shows risk increases in regions with higher percentages of Black or African American residents, and this finding is particularly noticeable in the South region. Some evidence exists that risk is also higher where there is a higher percent of smokers and a higher percent older population (light gray highlighted estimates). The testing variable was least consistent among the predictors in its effect on confirmed cases and deaths outcomes. It appears that more positive tests, both count of positive tests and percent positive tests, suggest more confirmed cases but not necessarily more deaths. Finally, all models include the same parameters for comparison, and they are well estimated at the 0.05 or 0.10 level for at least one model indicating an important relationship between that variable and the COVID-19 outcomes.

**Table 1:**
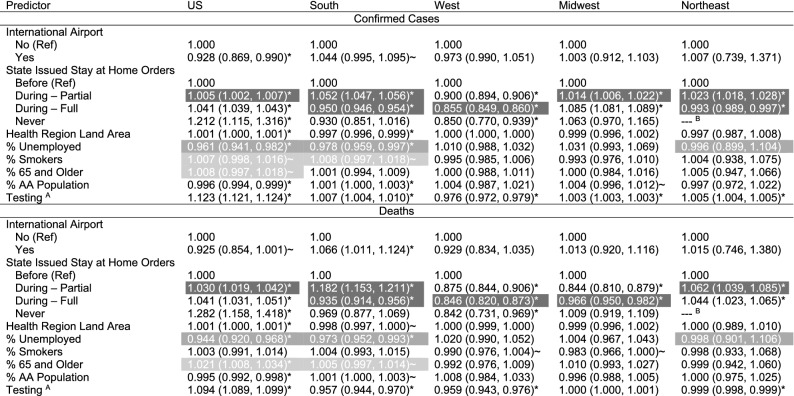
Fixed effect parameter estimates for the confirmed cases and deaths outcomes represented as inverse logarithm mean estimates and associated 95% credible intervals.

^A^Percent of positive tests was used for the Midwest and Northeast while log count of positive tests was used for all other models.

^B^There is no estimate associated with the never category of the stay at home orders variable for the NE model because all states in the Northeast issued a stay at home orders.

*Indicates well estimated Bayesian inference at the 0.05 level.

^~^Indicates borderline Bayesian inference.

**Figure 2:**
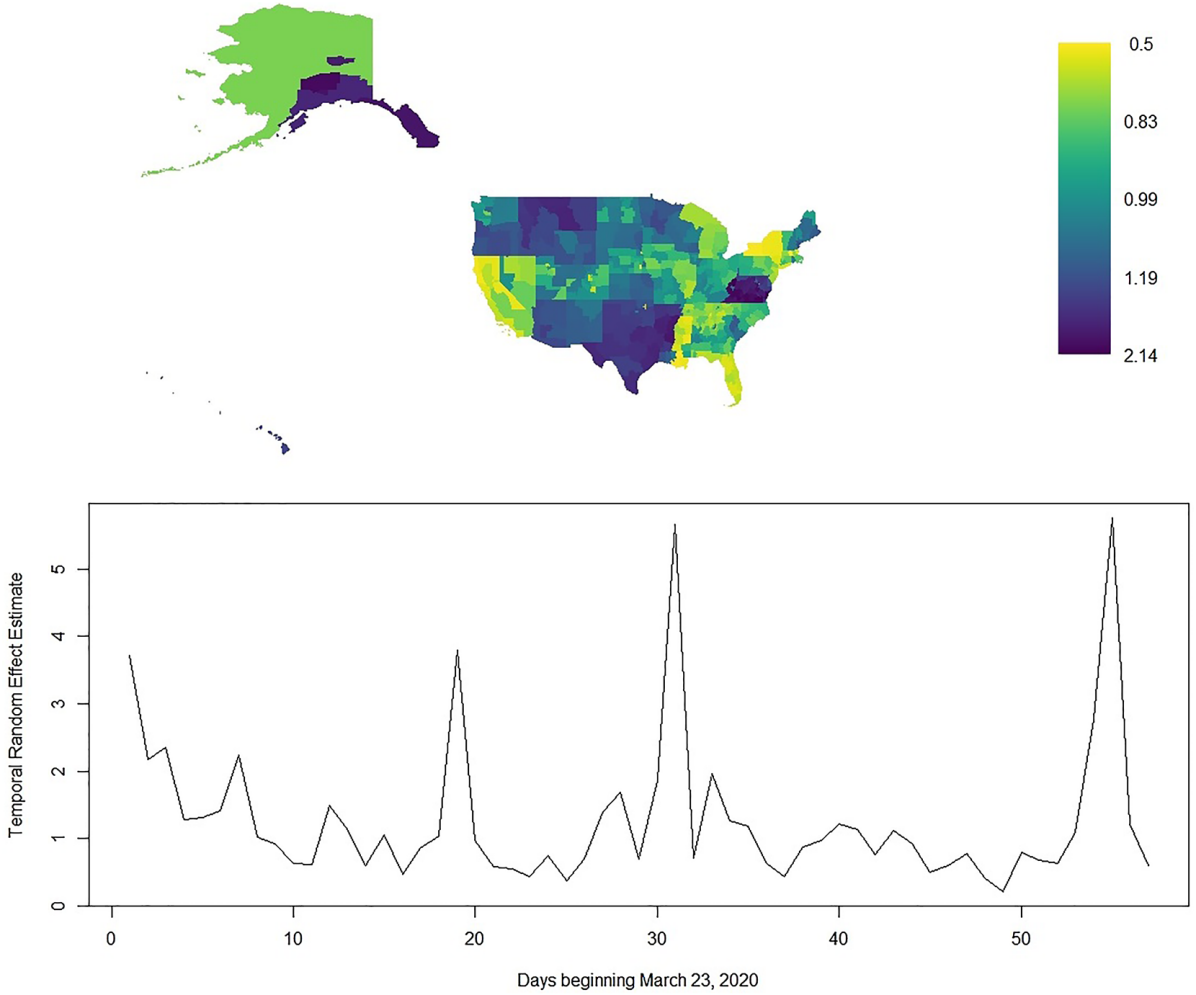
Spatial and temporal random effect estimates for the confirmed cases outcome considering all health regions in the United States.

Gray highlights indicate common estimate patterns mentioned in the text. Figure 2 displays the spatial and temporal random effect estimates for confirmed cases across the United States. These visuals display the residual spatial and temporal variation in the outcome after adjusting for the fixed effects in this table. Hence, increased (estimates greater than 1) or decreased (estimates less than 1) risk could arise from reasons pertaining to social distancing adherence or alternate population characteristics not considered in this study. In the spatial random effect, state borders are largely well defined here indicating consistency in the residual within states. After adjustment, many of the health regions with increased risk include parts of Idaho, Louisiana, Montana, and Alaska, Texas, Oklahoma, Arkansas, West Virginia, Virginia, Maryland, Delaware, and Hawaii. Alternatively, areas of decreased risk include parts of Louisiana and Mississippi, California, Florida, and New York. Finally, the temporal random effect parameter largely accounts for delays and errors in the data^11^. There are three sharp peaks at days 19 (April 10), 31 (April 22), and 55 (May 17).

## Discussion

The results presented here demonstrate the usefulness of these spatio-temporal statistical models for identifying several important risk factors for a range of COVID-19 outcomes. The use of Bayesian inference is both relevant to this inquiry and valuable as a benchmark and template for researchers to use for additional applications in data science. Additionally, the dashboards offer an ideal interactive environment for displaying these results for a range of interested stakeholders.

The two state-level spatio-temporal variables were among the most important fixed effects in the model, despite their different roles. The stay at home orders variable provided important predictive information to describe differences in risk for health regions under the four levels of the variable, while the testing variable served as a control variable for the number and positive rate of tests performed. For the stay at home orders predictor, all levels were well estimated for nearly all models, and there was general consistency across the models with respect to the direction of association for all models except, notably, the West model. Interestingly, these estimates show that there is generally less risk for health regions under full stay at home orders and health regions that were never under stay at home orders, and more risk for health regions under partial stay at home orders when compared to health regions before stay at home orders were implemented. It is possible that governors in states with full stay at home orders are appropriately maintaining these orders where health regions are at greater risk to maintain control of disease spread within their borders. For states with partial stay at home orders the findings suggest they may have prematurely removed restrictions or, perhaps it was a calculated risk and governors surmised that the hospital systems were prepared to accommodate the potential influx of cases. Less risk in states with partial or full stay at home orders could indicate that stay at home orders were successful and removal could be considered. Similarly, less risk for health regions that were never under stay at home orders could suggest that these governors made the appropriate decision for their states.

When considering results pertaining to testing, it is important to remember that these estimates do not suggest increased risk with more testing as a cause and effect relationship—merely that more comprehensive testing will necessarily increase the numbers of confirmed cases. Indeed, greater testing gives a better representation of the true disease presence in the country. Our examination time window and spatial aggregation may not be appropriate to examine the relationships between testing and the death outcomes. Examinations of these data in a recently published study suggest that there is a lag time of up to eight weeks between testing and death, potentially depending on the age of the individual^12^.

Other notable results uncovered here include decreased risk for health regions with more unemployment, increased risk for health regions as percent of Black or African American population increases in the South, and lower risk of new cases but higher risk of death for health regions with more older population. It is possible that with more unemployment, the frequency and volume of social interactions are diminished resulting in lower levels of disease transmission. Recall that these are unemployment estimates for March 2020, so health regions with more unemployment may also be health regions where more individuals were laid off or furloughed during the crisis. The finding that there is greater risk for health regions with more Black or African American population in the South aligns with anecdotal reporting and other findings regarding the disproportionate effects of this crisis on minority populations^13^. Incidentally, the South is an ideal place for the examination of minority populations because the presence is greater there than in other parts of the county. Finally, the age-related results—for regions with populations that have more residents aged 65 and older—suggest that older individuals adhere better to the social distancing standards but are still more susceptible to death, findings that reaffirm results in other studies^14,15^.

Although the fixed effect estimates were quite similar across models, the spatial and temporal random effects were different when the US Census-defined regions were compared to those generated for the entire United States. Some of these differences are a consequence of scaling the random effects in the different models, but several indicators suggest an opposite risk direction. This finding indicates that there are differences in the COVID-19 crisis across the country; thus, it is critical to consider these areas on their own. The US Census-defined region with the most consistent spatial random effect distribution to the one estimated for the United States as a whole was the Northeast. It is possible that the Northeast was driving much of the US-based model because the outbreak was centralized in that region for most of this time period. The West US Census-defined region was the most inconsistent with respect to the fixed effect estimates. We are not able to ascertain from these findings the exact cause of the disjuncture, but present two possible factors that could be explored in future academic inquiry: (1) the geographic and cultural heterogeneity within the West region and (2) the nature and duration of the stay at home orders in both California and Washington that appear different from other regions.

The statistical model implemented in this study has both advantages and disadvantages for understanding disease spread. First, this model appears robust to data delays that are inherent in real-time data^11^. For example, there is a strong seasonal trend in the data where more new cases are reported later in the week versus earlier in the week. However, that same trend is not evident in the temporal random effect estimates, and we observed no well estimated parameters when including a variable that attempted to adjust for this trend. The reason for the models’ robustness lies in the expected count (${e}_{ij}$) term, since that matrix by definition includes the same seasonal trend. One negative feature for these models involves the computational efficiency. While implementation via the INLA package in R speeds up the model fitting process, these complex models become more computationally inefficient with every day of new data generation. As such, real-time, or daily as used here, updates become less feasible the longer the crisis continues. Our current real-time rendering of these models uses weeks as the temporal unit for this reason. Finally, this is not a causal model, and we cannot determine the causes of higher or lower risk of COVID-19. Rather, we observe common characteristics among areas with higher or lower risk of COVID-19. While we include socio-demographic covariates, we cannot make claims of social determinants of health from our parameter estimates.

## Conclusion

In sum, this article demonstrates how statistical models of this nature and caliber can illuminate important relationships with disease spread and identify relevant population-level characteristics. Most importantly, we believe that these results confirm the benefit of state-issued stay at home orders as well as suggest compliance with respect to the directives towards the older population for adhering to social distancing guidelines.

## Materials and methods

### Data

Although most of the spatial data employed in this analysis are available at the county level, spatio-temporal statistical modeling of these data at this aggregation is complicated for two major reasons: (1) zero inflation and (2) large dimension. Both of these complications would render our methods inappropriate and inefficient. In order to avoid the issues associated with county-level modeling, we used a broader spatial aggregation—health regions, which are one or more counties serviced by the same health department^16^. By adopting health regions, the zero count of confirmed cases (as of May 18, 2020) dropped from 33,206 to 302 and the spatial dimension shrank from 3,142 (number of US counties) to 389 (number of US health regions). Correspondingly, we aggregated all county-level predictors and outcomes to the health region level. There is no evidence of loss of power with the aggregation of these data to the health region level^16^.

### Outcome measures

Outcome data were sourced from the Johns Hopkins University Center for Systems Science and Engineering^1^. Data for this study were geographically constrained to the United States and confined to the first eight weeks of recorded data (March 23, 2020 to May 18, 2020). Limiting the window of time to the first eight weeks was necessary and appropriate for three reasons. First, doing so guaranteed good representation in the two temporal varying predictors (state-issued stay at home orders and testing prevalence), particularly for the timeline pertaining to stay at home orders since several states had partially reopened but none had fully reopened. Second, by constraining the window of time it afforded the ability to examine data on a daily temporal scale as opposed to the real time models now that show data in weeks for computational efficiency. Finally, confining the data in this way allowed for the inclusion of testing information, which lags behind case counts in data release.

We examined four outcomes: number of confirmed cases, number of active cases, number of new cases, and number of deaths. Recovery data is not captured at the US county level and therefore is not incorporated as a stand-alone outcome or in the calculation of active cases. Additionally, there could be differences in covariate relationships based on the region of the country considered. As such, we made an estimation for the US as a whole and apportioned the numbers into the following regions as defined by the US Census Bureau^17^: South (S), West (W), Midwest (MW), and Northeast (NE).

### Covariates for adjustment

All models adjust for the same neighborhood-level covariates, which were selected based on two criteria: to describe the state-level responses to the pandemic and to represent population level characteristics that may contribute to the disparities in outcome counts between health regions. We elaborate on the selection of predictors in the paragraphs below. The statistical model indirectly adjusts for differences in population across the observed areas (see the Statistical model section below for more details). Images (gif or png) for all predictors considered are available for review in the supplemental files.

To understand and adjust for the state-level response to COVID-19, we considered two important temporal predictors, the state-by-state issuance of stay at home orders and the rate of testing. These variables are specified at the state-level because they are either mandated at the state-level or are only available at that level of aggregation, respectively. The self-constructed predictor representing presence of statewide stay at home orders required daily updates when running in real-time. The most current, convenient, and reliable information for this categorical variable (levels: before, during—partial, during—full, and never) came from the New York Times^7^. To ensure accuracy, we concurrently examined government mandates for more information. To construct the testing variable, we produced either a log transformation of positive test count or a percent of positive tests variable. The variable varied by US Census-defined region (US, S, W, MW, and NE) depending on what appeared best fitting according to goodness of fit criterion and most appropriate in terms of estimate directionality as a control variable^18^. The region-specific testing variable was consistent across outcomes within that region.

Population characteristics were also included as predictors in this model, specifically the presence of an international airport, land area of the health region, percent of the population that is unemployed, percent of the population that is a smoker, percent of the population that is aged 65 and older, and percent of the population that is Black or African American. The binary variable for presence of an international airport was used to represent community connectivity to international locations, which could be an important driver of disease progression particularly in this time window at the start of the pandemic. This predictor was created by combining information on airport locations from the U.S. Bureau of Transportation Statistics^4,5^ and international flights for each of those airports from the US Department of Transportation.

Whereas urban areas typically comprise small health regions, rural areas are found in larger health regions. Size of a health region necessarily influences important factors like access to care, access to quality care, and capacity of health providers. Therefore, we adjusted for urbanicity by including information on the land area of each observation. Unemployment was estimated as the percent of the population that was unemployed in March 2020 in each health region using information from the Bureau of Labor Statistics^6^. The rest of the population demographic variables—smoking, age, and race—are 2018 estimates from the US Census Bureau and were included as proxies for comorbidities and vulnerable populations^3,19^.

### Statistical model

The statistical model applied here is commonly used in disease mapping for aggregated count outcomes, and is a variation of a Bayesian spatio-temporal Poisson Knorr–Held model^8–10,20–22^. This model can be defined as follows for health region $i$ and day $j$:

$${y}_{ij}\sim Pois\left({\mu }_{ij}\right)$$

$${\mu }_{ij}={e}_{ij}{\theta }_{ij}$$

$$\mathrm{log}\left({\theta }_{ij}\right)={X}_{ij}^{\mathrm{{'}}}\boldsymbol{\beta }+{u}_{i}+{\gamma }_{j}$$

where ${y}_{ij}$ is the outcome of interest (count of confirmed cases, active cases, new cases, or deaths), ${\mu }_{ij}$ is the mean of the Poisson model, ${e}_{ij}$ is the expected count, ${\theta }_{ij}$ is the relative risk, ${X}_{ij}^{\mathrm{{'}}}$ is the design matrix for the predictors, $\boldsymbol{\beta }$ is the vector of fixed effect estimates, ${u}_{i}$ is the spatial random effect, and ${\gamma }_{j}$ is the temporal random effect. ${e}_{ij}$ is calculated as the rate of infection across all health regions on a given day times the population at risk for a given health region, which is assumed constant over time. As such, a unique ${e}_{ij}$ is produced for each health region and day. Given the Bayesian methodology applied here, prior distributions were required for all parameters and they were defined as follows: ${\beta }_{k}\sim N\left(0,{\tau }_{\beta }^{-1}\right)$ for each of the $k$ fixed effect parameter estimates, ${u}_{i}\sim N\left(0,{\tau }_{u}^{-1}\right)$ for an uncorrelated spatial random effect, ${\gamma }_{j}\sim N\left({\gamma }_{j-1},{\tau }_{\gamma }^{-1}\right)$ for a temporal random walk effect, and all precisions were such that $\tau \sim Gam\left(\mathrm{2,1}\right)$. All these prior distributions are considered non- or weakly-informative and sensitivity analysis (data not shown) suggests little influence on the resulting parameter estimates. This model description applies to all outcomes (confirmed cases, active cases, new cases, and deaths) and US Census-defined regions (US, S, W, MW, and NE) considered.

### Computational details

R statistical software furnished much of the means for data processing and analysis in the work presented here. Specifically, the R packages rgdal, INLA, and fillmap were necessary for spatial data processing, statistical modeling, and spatial plotting respectively^23–32^. We, along with several others from the UNCW Data Science program, produced data visualization dashboards with an array of popular software including R Shiny, Tableau, and Power BI^2^. In addition to the regression model results, these dashboards also display general spatial, temporal, and spatio-temporal tracking of COVID-19 cases and deaths at the county, state, national, and international levels. Code for the statistical models and Shiny apps are available at this GitHub repository.

## Supplementary Information

Supplementary Information.
